# Advances in Probing Amyloid Heterogeneity Using Vibrational Spectroscopy and Imaging

**DOI:** 10.1021/acs.jpcb.5c06431

**Published:** 2025-11-14

**Authors:** Cade K. Rohler, Kayla A. Hess, Lauren E. Buchanan

**Affiliations:** Department of Chemistry, Vanderbilt University, Nashville, Tennessee, 37235, United States

## Abstract

The misfolding and aggregation of proteins into amyloid fibrils are associated with numerous human diseases; however, our understanding of the mechanisms by which amyloid proteins exert their toxicity remains limited. This gap in knowledge can largely be attributed to the significant polymorphism of species that form during aggregation, ranging from short-lived soluble oligomers to various polymorphic fibrils, compounded by the complex interplay of other proteins and biomolecules. Vibrational spectroscopies are particularly well-suited for studying these heterogeneous mixtures and, with the integration of site-specific probes, can provide residue-level structural information. This perspective highlights recent advances in the application of Raman and infrared (IR) spectroscopy and imaging techniques to elucidate aggregation mechanisms, characterize oligomer and fibril structures, analyze plaque compositions, and investigate the effects of coassembly and cross-seeding. These efforts move us toward a greater understanding of how amyloids form under disease-relevant conditions, which may provide new routes toward targeted therapeutics.

## Graphical abstract

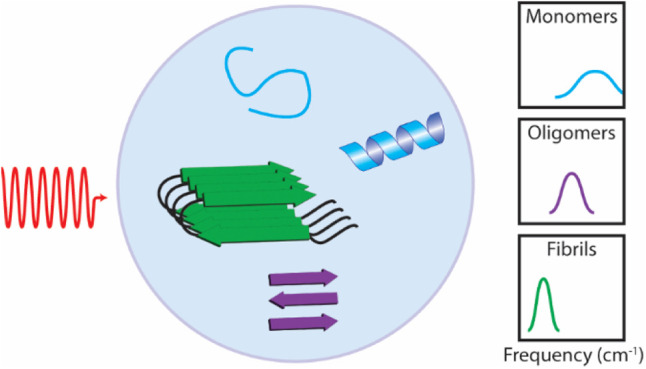

## Introduction

The misfolding of proteins into amyloid fibrils is implicated in more than 30 human diseases, including Alzheimer’s disease (AD), Parkinson’s disease, and type II diabetes.^1,2^ Additionally, numerous examples of functional amyloids, in which fibrils are involved in natural biological functions, have been identified, and studies suggest that many proteins may have the intrinsic ability to form amyloid fibrils given the proper conditions.^1,3,4^ While the sequences and native conformations of amyloidogenic proteins vary, amyloid fibrils are characterized by a shared cross-β structural motif in which extended β-sheets stack with their strands aligned perpendicular to the fibril axis.^1^ Most evidence points to short-lived oligomeric aggregates as the primary pathogenic species in amyloid disease, although mature fibrils can still exhibit considerable toxicity.^1,2,5–8^ Thus, it is essential to characterize the species involved throughout the entire aggregation pathway. A further complication in our understanding of amyloid disease is the high degree of polymorphism found in both synthetic and patient-derived samples. Amyloid fibrils aggregated under *in vitro* conditions are highly sensitive to aggregation conditions, including solvent, pH, or temperature.^9–13^ This polymorphism extends to human-derived samples, where fibrils isolated from patients with distinct disease presentations have been found to differ in morphology.^11–15^ Oligomers are likely to display similar heterogeneity, although multiple polymorphs may be derived from a common oligomeric state.^16^ As fibril morphology also differs significantly between *in vitro* and *ex vivo* samples, research is currently attempting to consolidate the results from each study type by moving toward conditions that more closely mimic those within the body and advancing techniques to enable this.

While the cross-β structures inherent to amyloid fibrils are determined via X-ray diffraction,^17^ additional techniques are needed to characterize morphological differences. Amyloid polymorphism is often identified via transmission electron microscopy (TEM) and solid-state nuclear magnetic resonance (ssNMR). TEM allows fibrils to be imaged with sufficient spatial resolution to characterize differences in supramolecular morphology, such as alterations in fibril width and twist periodicity.^9,17^ Additionally, dark-field electron microscopy can identify changes in the mass-per-length values, indicating how many cross-β subunits make up a fibril.^9^ However, these techniques do not have the structural resolution necessary to recognize changes in conformation between the backbones, side chains, or different β-strands. Instead, ssNMR is typically utilized to identify such features and thus construct structural models of amyloids.^17–19^ In recent years, cryo-electron microscopy (cryo-EM) has emerged as a powerful alternative for determining the core structure of amyloid fibrils.^20,21^ Each of these techniques provides great insight into amyloid polymorphism on a structural level but often requires the use of static, highly-ordered, homogeneous samples, limiting their ability to track structural changes over the course of aggregation. Instead, fluorescence assays with dyes such as thioflavin T (ThT) are typically used to monitor amyloid aggregation kinetics, but provide very limited structural information.^22^ Unlike these standard approaches, vibrational spectroscopy can readily provide simultaneous spatial and temporal information.^16,23,24^

Even in their most fundamental implementation, both infrared (IR) and Raman spectroscopy are capable of detecting heterogeneities in samples of amyloid proteins. A particular strength of these optical spectroscopies compared to other biophysical techniques is their ability to analyze proteins in a wide variety of states, ranging from soluble monomers and oligomers to insoluble fibrils, and from amorphous aggregates to highly ordered crystals. In fact, it has been hypothesized that the versatility of IR spectroscopy may have contributed to the discrepancy between early Fourier transform IR (FTIR) studies and later NMR characterization of amyloid fibrils.^25^ As early as the 1970s, results from FTIR indicated that amyloid samples had significant antiparallel β-sheet content, but nearly all modern high-resolution structural models indicate that fibrils comprise primarily parallel β-sheets. As we have come to realize the importance of transient oligomeric species in the pathology of amyloid disease, many have suggested that antiparallel intermediates may dominate at early stages before converting to the final fibrillar structure.^26–28^ While these species would be absent in the highly processed, homogeneous samples required for NMR and X-ray crystallography, vibrational spectroscopies can observe the evolution of such structures in real time.

Many variations of IR and Raman spectroscopy have been used to study amyloid proteins. Great progress has been made in the development of label-free methods for differentiating spectral contributions in heterogeneous mixtures. Vibrational labels can be incorporated to track structural changes with single-residue resolution or differentiate proteins of interest in more complex biological samples. Vibrational spectroscopies have been coupled with imaging modalities to gain spatial resolution, revealing structural variations even within individual aggregates. In this perspective, we highlight recent advances in the use of vibrational spectroscopy and imaging for the characterization of amyloid heterogeneity.

## Vibrational Spectroscopy

Vibrational spectra are rich with information about both the backbone and side chains of proteins. Extensive reviews on IR and Raman spectroscopy of proteins exist,^29,30^ to which we refer the reader for a more in-depth discussion of their spectral analysis. Most studies of amyloid proteins focus on the backbone amide modes (Figure 1A), although side chain modes can appear within the same spectral regions and interfere with or even couple to backbone vibrations.^31^ Of the amide modes, amide I is most commonly used in both IR and Raman spectroscopy to monitor changes in secondary structure (Figure 1B). Arising primarily from the C=O stretch, it produces strong signatures in IR and Raman spectra in the 1600–1700 cm^–1^ region and is highly sensitive to vibrational coupling, hydrogen bonding, and the local environment of the backbone carbonyls. In aqueous solution, disordered and α-helical structures display overlapping peaks centered between 1640 and 1660 cm^–1^ for both techniques. β-sheets produce two features: an E_1_ mode (1620–1635 cm^–1^), with a net dipole perpendicular to the β-strands and an A mode (1670–1685 cm^–1^) with a net dipole parallel to the strands. Due to the symmetry of the modes and their respective dipole strengths (IR) and polarizabilities (Raman), the lower frequency E_1_ mode is primarily observed in IR spectra (with a weak A mode present for antiparallel β-sheets), while the A mode is more dominant in Raman spectra. For amyloid proteins, the frequency differences of the amide I mode can be used to detect structural heterogeneities. For example, recent work by Dec et al. sought to understand how adenosine triphosphate (ATP) triggers fibrillization of chimeric peptides formed by fusing a highly amyloidogenic fragment of insulin with oligolysine chains of various lengths.^32^ Using AFM and optical microscopy, they observed the aggregation process and found that increasing the length of the flexible oligolysine chain resulted in liquid–liquid phase separation (LLPS) prior to fibrillization. Furthermore, fibril polymorphs were observed within each sample, but no conclusions could be drawn from imaging on the influence of the oligolysine length on morphology. However, FTIR revealed a gradual blueshift in β-sheet frequency from 1622 to 1635 cm^–1^ as the length of the oligolysine chain increased from 8 to 40 residues, with the additional appearance of a peak at 1651 cm^–1^ corresponding to increased disordered structures for the longest peptides (Figure 1C). They suggest that the longer chimeric proteins behave similarly to tau, which also undergoes LLPS and forms fibrils in which less ordered segments form “fuzzy coats”^33^ that are difficult to observe in AFM or TEM but can be detected with IR spectroscopy.

**Figure 1:**
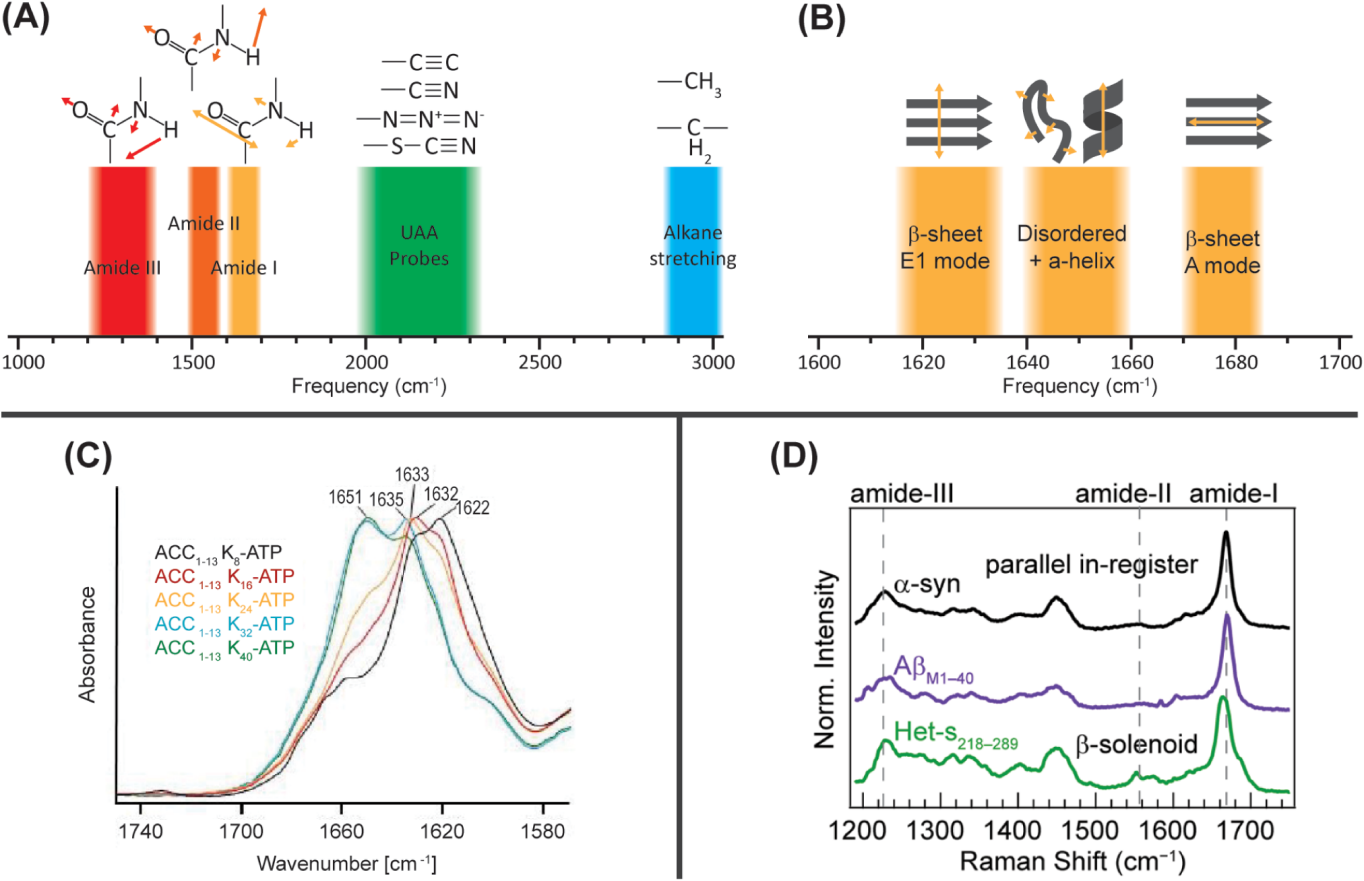
Vibrational spectroscopy of proteins: (A) Approximate frequency ranges for the major vibrational bands of proteins. (B) The amide I range expanded to show typical frequency ranges of secondary structures. (C) FTIR spectra of chimeric peptide ACC_1–13_K_n_-ATP revealing a blueshift in the amide I frequency as the length of the oligolysine chain increased. Reproduced from ref 32. Copyright 2023 American Chemical Society. (D) Raman spectra in the amide fingerprint region of α-synuclein (black), Aβ_M1–40_ (purple), and Het-s_218–289_ (green) with relevant amide modes indicated by dashed lines. Reproduced from ref 34. Available under a CC BY 4.0 license. Copyright 2024 Ramos and Lee.

The amide II (1480–1580 cm^–1^) and amide III (1200–1400 cm^–1^) bands both arise primarily from combinations of NH bending and CN stretching motions. These modes are generally more difficult to interpret and correlate directly to protein structure than the amide I. However, it has been suggested that the fine structure of the amide III band may be more sensitive to conformational differences than the amide I and can be used to “fingerprint” amyloid structures in Raman spectra. Flynn et al. demonstrated this capability by comparing the Raman spectra of three pathological amyloids and two functional amyloids.^35^ They found that spontaneous Raman spectroscopy is not only capable of distinguishing distinct structural conformations, such as in-register parallel β-sheets versus β-solenoids, but can also differentiate between proteins that adopt similar conformations. For example, *N*-acetyl α-synuclein (*N*-acetyl α-syn) and amyloid-β 1–40 (Aβ_1–40_) both form fibrils with similar morphologies and in-register parallel β-sheets. While their amide I bands are essentially indistinguishable, the amide III bands are distinct due to differences in their supersecondary structural motifs (Figure 1D).^34,35^ Harper et al. have expanded upon this fingerprinting by showing that structural models for different amyloid polymorphs can be built based on Raman spectra.^36^ Capitalizing on the sensitivity of the amide III band, they were able to extract dihedral angles from their spectra to use as structural constraints for molecular dynamics simulations. These models can serve as a guide to design additional experiments with higher structural resolution, which would allow the structures to be refined further.

## Resolving Structural Polymorphs within a Sample

While it is clear that vibrational spectroscopies are highly sensitive to differences in amyloid structure, it can be challenging to separate spectral signatures that arise from different structures. These structures can include monomers, oligomers, and fibrils at different stages of aggregation, as well as different polymorphs of each aggregate type. In this section, we highlight three methods that have recently been developed to help differentiate between the variety of aggregates that can coexist within a single sample.

### IR-DOSY

While it is possible to propagate and isolate a single fibril morphology for structural characterization, it is far more challenging to isolate the transient oligomeric species thought to represent the primary pathogenic species in amyloid diseases. A handful of oligomers have been successfully stabilized, but their preparation requires stringent conditions that may not accurately reflect what occurs in biology.^41,42^ As an alternative, recent work by Giubertoni and coworkers has developed an approach for separating species based on size for *in situ* spectroscopic analysis. Inspired by the NMR field, they developed infrared diffusion-ordered spectroscopy (IR-DOSY).^37^ Utilizing a flow cell with two injection ports, the sample mixture enters the bottom half of the cell, and pure solvent enters the top half. After the flow is stopped, molecules within the sample mixture passively diffuse into the pure solvent region, through which the detection beam passes. As the diffusion coefficient of a molecule is related to its size according to the Stokes–Einstein relation, time-dependent IR spectra can be used to resolve spectral features arising from molecules with different sizes (Figure 2A). Further, the size of each species can be calculated directly from the diffusion coefficient. The initial demonstration focused on separating signals from small molecules that could potentially interfere with protein signals; these could include naturally occurring small molecules from biological samples, such as glucuronic acid, or residual species from synthesis and purification processes, such as trifluoroacetic acid (Figure 2B).

**Figure 2:**
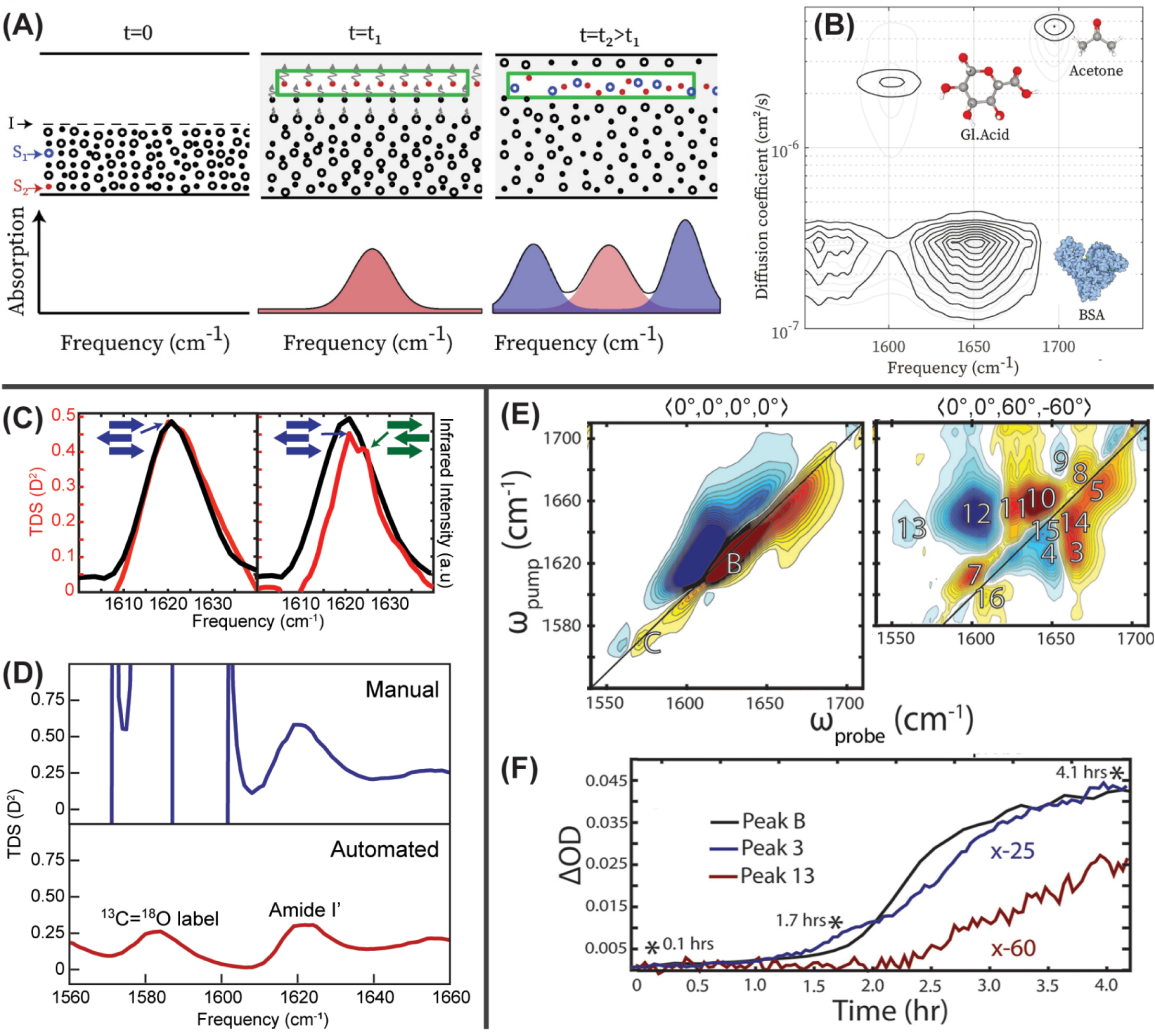
Methods of resolving structural heterogeneities in label-free vibrational spectra: (A) Representation of multianalyte diffusion and corresponding absorption spectra over time with an IR-DOSY setup. (B) IR-DOSY spectrum showing the separation of bovine serum albumin from small molecule cofactors. Panels A–B reproduced from ref 37. Available under a CC BY 4.0 license. Copyright 2022 Giubertoni et al. (C) Comparison of 2D IR diagonal traces (black) and calculated TDS spectra (red) for KFE8 (left) and Ac-KFE8 (right), highlighting how TDS can detect differences in β-strand alignments that are invisible to 2D IR. Adapted from ref 38. Copyright 2022 American Chemical Society. (D) Comparison of calculated TDS spectra for ^13^C^18^O-labeled amylin without manual (blue) vs automated (red) background correction. Data originally published by Boutwell et al.^39^ (E) 2D IR spectra of amylin collected with ⟨0°,0°,0°,0°⟩ polarization (left) and ⟨0°,0°,60°,–60°⟩ polarization (right) reveal cross peaks concealed by strong on-diagonal amide I signal. (F) Intensity of diagonal β-sheet peak B (black), crosspeak 3 (blue), and crosspeak 13 (red) over the course of amylin aggregation. While crosspeak 3 kinetics matches the main β-sheet peak, the delayed kinetics of crosspeak 13 supports a secondary nucleation model. Panels E–F reproduced from ref 40. Copyright 2023 American Chemical Society.

In a follow-up study, however, Giubertoni et al. demonstrated effective separation of signals arising from either monomers or amyloid fibrils of bovine serum albumin.^43^ Species as large as amyloid fibrils diffuse too slowly to be observed on reasonable time scales, so they adapted the IR-DOSY technique to measure depletion of signals from the sample-filled region rather than their arrival in the solvent region. By allowing the signal of the monomer to decay to 50% of its original value, indicating complete diffusion across the solution, a difference spectrum of the mixture at 100% and 50% monomer signals was calculated to obtain the spectral contribution that came from only the amyloid component. While the diffusion coefficient, and thus the size, of the fibrils themselves cannot be determined due to their lack of diffusion, it is still possible to do so for the smaller species in the mixture. Ultimately, IR-DOSY may prove to be a powerful approach for resolving the wide variety of aggregated species present in amyloids, allowing both the size and secondary structure of each species to be ascertained simultaneously. However, the transient nature of prefibrillar oligomers will likely present a significant challenge. As smaller species such as monomers and oligomers diffuse, they will continue to aggregate. The formation and evolution of oligomeric species will convolute the straightforward calculation of diffusion coefficients from time-dependent IR spectra and will thus require more extensive kinetic modeling to isolate spectral features arising from these species.

### Transition Dipole Strength Analysis

Most IR and Raman investigations of proteins rely on correlating the amide frequency to structure. The amide modes shift in frequency due to changes in vibrational coupling between amide groups as the proteins adopt ordered secondary structures, resulting in the characteristic frequency ranges for amide I discussed previously. However, vibrational coupling also redistributes oscillator strengths, which leads to changes in the transition dipole strength (TDS, μ). As the TDS of a vibrational mode is directly related to its extinction coefficient, the intensity of an IR peak should serve as another metric for vibrational coupling and, thus, structural ordering. However, linear absorption techniques, such as FTIR, are insensitive to coupling-induced changes in TDS because the integrated peak areas remain constant.

In contrast, the integrated peak areas in two-dimensional infrared (2D IR) spectroscopy are highly sensitive to changes in TDS. While linear absorption spectra scale as ${|\mu |}^{2}$ , 2D IR spectra scale as ${|\mu |}^{4}$ . Grechko and Zanni utilized this difference to develop a method of measuring the absolute TDS based on ratios of 2D IR to linear IR signals.^44^ They proposed that TDS measurements can provide a more sensitive measure of vibrational coupling than frequency alone, particularly when variations in coupling do not produce measurable frequency shifts.^44–46^ Recent work by our group has demonstrated the broader potential of TDS analysis as a label-free approach to reveal differences in secondary structure between protein aggregates that appear homogeneous by most other techniques. For example, 2D IR spectra of an octapeptide (KFE8) and its acetylated analogue (AcKFE8) are identical, despite the variants forming aggregates with different morphologies. However, TDS spectra revealed two distinct peaks underlying the vibrational transition for AcKFE8 (Figure 2C).^38^ Based on additional studies with isotope-labeled peptides,^47^ we were able to assign these TDS features to antiparallel β-sheets that differ by a two-residue shift in the register of the β-strands. In contrast, TDS spectra of KFE8 showed only a single peak that aligned with one of the AcKFE8 features, which suggests that the second strand alignment underlies the altered morphology of the acetylated aggregates. Further, we showed that TDS spectra can be obtained with the same temporal resolution as standard 2D IR spectra, enabling the detection of oligomeric species during early stages of aggregation that may prove critical to understanding the molecular origin of amyloid diseases.

TDS analysis has proven similarly useful in characterizing differences in the aggregation dynamics of insulin variants^48^ and determining how gold nanoparticles alter the structure of human amylin fibrils.^49^ However, the adoption of TDS analysis has remained limited due to issues with precision for a single measurement and challenges in applying it to weaker signals. While some variation is expected for amyloidogenic samples due to their inherent heterogeneity,^46^ we have observed sample-to-sample variations even for small molecules (although averaging the TDS over multiple measurements ultimately yields the correct value). Further, the need to take ratios of 2D to linear IR signals leads to large artifacts when the signal-to-noise ratio is low. We found that most of these limitations arise from the manual correction of the linear IR baseline. To address this limitation, we have demonstrated that a machine learning algorithm can be used to correct signal backgrounds and improve the precision of TDS measurements from 20 to 30% error to 3%, on average (Figure 2D).^39^ It also enabled TDS analysis to be applied to much weaker signals, such as individual amide bonds, which can be tracked throughout the aggregation process to provide a more precise measure of structural order at the single-residue level.

### Crosspeak Analysis

As a vibrational analogue of 2D nuclear magnetic resonance spectroscopy, 2D IR has many additional advantages over traditional linear IR and Raman techniques beyond enhanced sensitivity to changes in TDS. Some of these advantages include improved resolution of congested peaks, the ability to measure spectral diffusion, and the observation of cross peaks.^50,51^ The intensity and time dependence of these cross peaks can report on coupling, chemical exchange, or energy transfer between two vibrational modes. As with all nonlinear spectroscopic techniques, the polarization of each pulse in the 2D IR pulse sequence strongly affects the emitted signal and can be used to determine the angle between coupled modes and suppress or enhance specific spectral features.^51^ One of these polarization schemes is capable of eliminating the strong diagonal peaks from a 2D IR spectrum, which often obscure the much weaker crosspeaks.^52^ When Farrell and coworkers applied this polarization scheme to human amylin, they identified 22 new crosspeaks that appear at different stages of fibril formation (Figure 2E).^40^ As vibrational coupling is directly related to molecular structure, these crosspeaks can provide another sensitive measure of the protein secondary structure. Based on the kinetics, they were able to group the crosspeaks into two subsets that correspond to two distinct fibril polymorphs, which form at different rates (Figure 2F). Kinetic modeling supported the slower polymorph as a novel fibril structure that forms via secondary nucleation off the faster-forming polymorph. Thus, crosspeak analysis provides another label-free method of resolving polymorph structures and defining aggregation mechanisms.

## Site-Specific Probes for Increased Structural Resolution

While advances in the implementation of vibrational spectroscopies have significantly enhanced their ability to resolve and characterize the variety of aggregate states and structural polymorphs inherent in amyloid samples, even greater structural detail can be obtained via the incorporation of site-specific probes. Isotope substitution is perhaps the ideal method for incorporating vibrational probes, as heavy atom labeling of the backbone amides is minimally perturbative to protein structure and dynamics. While it is straightforward to incorporate single isotope-labeled residues in synthetic peptides,^53^ longer proteins generally must be produced via expression in cells, which typically limits isotope labeling to uniform labeling of either the full protein^31,54^ or a continuous segment that can subsequently be assembled into the full-length protein via ligation.^55,56^ As an alternative to isotope labeling, unnatural amino acids (UAAs) with IR- or Raman-active functional groups can be incorporated into expressed proteins via site-directed mutagenesis. In this section, we review both labeling approaches.

### Isotope Labeling

As the amide I mode primarily comprises carbonyl stretching,^13^C- or ^13^C^18^O-labeling is most commonly employed to produce a redshift of approximately 40 cm^–1^ or 55 cm^–1^, respectively. Site-specific isotope labeling has been widely used in FTIR and 2D IR spectroscopy to probe the residue-level structure and dynamics of amyloid peptides.^62,63^

The power of isotope labeling in conjunction with vibrational spectroscopy for understanding amyloid aggregation is perhaps best illustrated by its successful use in developing a detailed aggregation pathway for human amylin fibrils. In a series of publications spanning over a decade, the Zanni group has used 2D IR spectroscopy to resolve specific molecular contacts throughout the aggregation of amylin (Figure 3A).^16,57–60^ In the earlier studies, single ^13^C^18^O labels were used to detect the formation of in-register parallel β-sheets; as β-sheets form and the isotope-labeled residues align, vibrational coupling further redshifts the ^13^C^18^O-labeled amide I peak. Using this approach, they were able to identify a novel oligomer with parallel β-sheet structure at residues 23–27 that forms during the lag phase of amylin aggregation^57^ and show that it serves as a common intermediate for two fibril polymorphs.^16^ While single labels are highly effective for monitoring the intermolecular contacts that form between strands in amyloid β-sheets, they are unable to probe intramolecular contacts such as those in α-helices. To address this gap, the Zanni group developed 2D IR dihedral indexing, which employs pairs of ^13^C^18^O at neighboring residues to detect changes in dihedral angles,^60^ similar to the chemical shift index used in NMR spectroscopy for assigning protein secondary structure. Relative to a fully disordered protein, a blueshift with an intensity increase for the labeled amide I mode indicates that labeled carbonyls are oriented parallel to each other and thus must participate in a helical structure; in contrast, a redshift with an intensity increase indicates that the carbonyls are antiparallel and must be part of a β-strand. Using this approach, they discovered that while amylin always forms a transient parallel β-sheet at residues 23–27 during the lag phase, the structure near the N-terminus is dependent on the environment. In buffer, residues 12–13 also adopt a β-sheet structure during the lag phase; in lipid vesicles, however, these residues adopt an α-helical structure.^58^

**Figure 3:**
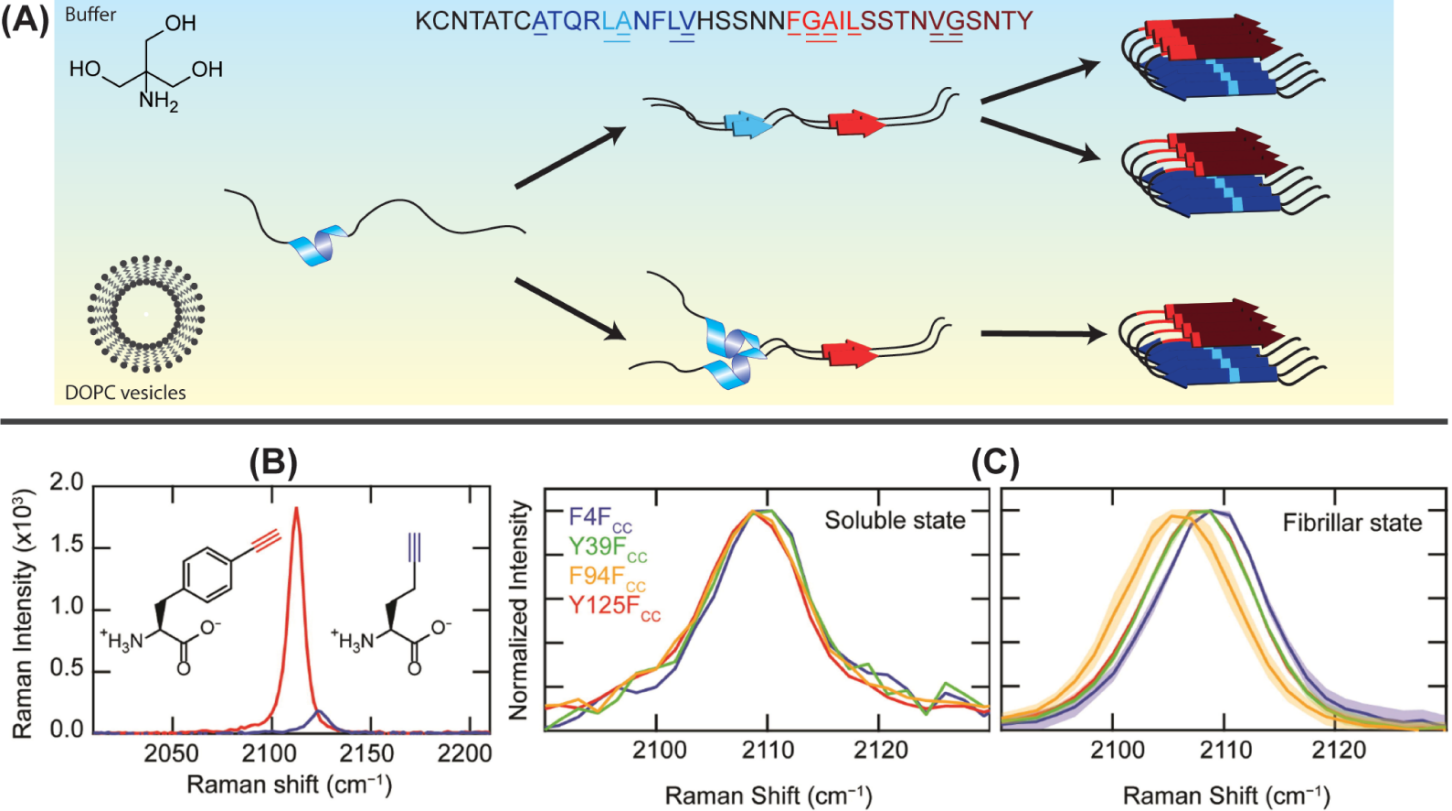
Site-specific probes of amyloid structure: (A) Illustration of amylin aggregation pathways in buffer (blue) and vesicles (yellow) summarizing results from refs 16 and 57–60. Sites for single and double ^13^C^18^O isotope labels are indicated by underlined sections in the sequence. While monomers adopt the same structure in both buffer and vesicles, different oligomeric structures are observed. In buffer, two fibril polymorphs both originate from the same oligomeric state. (B) Alkyne stretching bands in Raman spectra of F_cc_ (red) and HPG (blue), highlighting the aromatic enhancement in F_cc_. (C) F_cc_ labels incorporated at positions F4 (purple), Y39 (green), F94 (orange), and Y125 (red) undergo site-specific frequency shifts as α-syn transitions from soluble monomers (left) to fibrils (right). Panels B–C reproduced from ref 61. Available under a CC BY-NC 4.0 license. Copyright 2023 Watson and Lee.

Where the Zanni group found that amylin forms different oligomeric structures depending on the aggregation environment, Vosough et al. discovered that the structure of Aβ_42_ oligomers can change with their size.^64^ Using FTIR spectroscopy, they observed the progression from monomeric to small (∼60 kDa) oligomers and subsequently to larger (∼100 kDa) oligomers in an aqueous buffer. Both oligomeric states contained antiparallel β-sheets, but the incorporation of site-specific ^13^C labels revealed that residues A30 and I32 participate in early β-sheets, while residues V18 and F20 remain disordered until later in the aggregation process, when larger oligomers are formed. Double ^13^C-labeling was used to detect intra- and intermolecular contacts, which allowed them to determine that Aβ_42_ adopts a β-hairpin structure exclusively in the larger oligomer. Notably, the addition of sodium dodecyl sulfate did not produce a new oligomer structure but instead appeared to stabilize the smaller oligomers.

It is important to note that vibrational modes within amino acid side chains can appear in the same spectral region as the isotope-labeled amide I. These side chains can interfere with the interpretation of isotope-labeled peaks, although this presents more of a problem when studying helical peptides than the extended β-sheet structures of amyloid proteins. While such residues can be mutated to remove the interference, such mutations can alter the protein structure and dynamics. Alternatively, we have shown that extending the delay between pump and probe pulses in 2D IR to ∼600 fs suppresses side chain signals while retaining sufficient amide I signal for both native and isotopically labeled residues.^65^

### Unnatural Amino Acids

Site-specific isotope labeling is not always feasible, especially for longer proteins that cannot be produced by solid-phase peptide synthesis. One of the most promising alternatives for site-specific labeling is the incorporation of UAAs with modified side chains containing nonnative functional groups. Ideally, these functional groups are small, minimally perturbative, and have vibrational modes that fall within the cell-silent region of the vibrational spectrum (∼1800–2800 cm^–1^). Some of the most common UAA probes that meet these criteria include alkyne, nitrile, azide, or thiocyanate groups (Figure 1A).^66^ Alkynes have a strong Raman cross-section which can be further increased by conjugation to an aryl ring, as Watson and Lee recently demonstrated in their use of 4-ethynyl-l-phenylalanine (F_cc_) to monitor the cellular uptake of α-syn fibrils (Figure 3B).^61^ Structural perturbation was minimized by inserting F_cc_ at positions that have aromatic side chains in the native sequence, although UAAs with aliphatic side chains, such as homopropargylglycine, can be used if such sites are limited. Watson and Lee demonstrated that F_CC_ exhibits site-specific shifts of the alkyne stretching mode upon the aggregation of α-syn. Further, by monitoring the alkynyl mode, they observed changes in the local environment and structural remodeling as fibrils are internalized into human neuronal cells (Figure 3C). UAAs have also been used in linear and 2D IR protein studies,^67^ although with few direct applications to amyloid proteins.^68^ However, UAAs provide a unique opportunity for IR spectroscopy to probe protein structure in a spectral region that has a minimal water background.

## Vibrational Imaging

While we have shown that recent advances in vibrational spectroscopies have enabled the characterization of heterogeneous structures in bulk samples, it can still be challenging to ascertain the level of polymorphism and match spectral signatures with specific aggregate morphologies. By coupling vibrational spectroscopy with an imaging modality, we can spatially resolve the spectral information and correlate the molecular structure with supramolecular morphology. Optical microscopies have been used extensively for vibrational imaging of amyloid fibrils due to their accessibility, although probe-based techniques have become increasingly popular due to their higher spatial resolution. These techniques hold great promise for providing unprecedented insights into complex phenomena that are challenging to investigate using conventional solution-based spectroscopy.

## Optical Microscopy

Traditionally, vibrational imaging is implemented with either confocal or widefield microscopy. The spatial resolution of these techniques is inherently diffraction-limited to approximately half the wavelength of the light used in the experiment. This corresponds to a spatial resolution of 200–300 nm for Raman-based techniques, while IR-based techniques are limited to the micron scale. Raman imaging also requires minimal sample processing and, unlike IR-based techniques, does not suffer from water absorption. Thus, it is uniquely suited for imaging amyloid aggregates across a wide range of sample conditions, including *ex vivo* tissues.^69,71–75^ As a result, Raman spectral imaging (RSI) is the most common form of vibrational microscopy, although optical photothermal IR (OPTIR) imaging is emerging as a novel approach to circumvent the IR diffraction limit.

### Raman Spectral Imaging

First demonstrated in the 1970s, RSI is relatively straightforward to implement—in its most basic form, requiring only a confocal microscope, a monochromator, and a charge-coupled device (CCD) camera. While more advanced Raman-based imaging techniques have been developed, including nonlinear techniques such as stimulated Raman spectroscopy (SRS)^71–73^ and coherent anti-Stokes Raman spectroscopy (CARS),^74,75^ confocal Raman imaging remains a cost-effective, label-free tool for the identification of amyloid aggregates within *ex vivo* tissues. Mrđenović and coworkers used hyperspectral Raman imaging to examine plaques within brain slices from transgenic arcAβ mice, a model for Alzheimer’s disease (Figure 4A).^69^ Hyperspectral imaging allowed them to quantify signals arising from nucleic acids, lipids, amyloid aggregates, and other proteins within each plaque. They found that the lipid and protein contents can vary significantly, suggesting that chemical composition may vary between different types of plaques. Work by the Lee group has advanced the use of vibrational probes in RSI for increased structural resolution.^34^ As discussed in the section on site-specific probes, isotope substitution serves as a nonperturbative method of labeling vibrational modes. While RSI generally lacks the sensitivity to observe site-specific isotope labels within the crowded amide I spectral region, segmental ^13^C-labeling of α-syn allowed them to determine which polypeptide region first adopts a β-sheet structure.^56^ Alternatively, UAAs containing terminal alkynes, such as 4-ethynyl-l-phenylalanine (F_CC_), contain Raman-active functional groups that appear within the cell-silent region of the Raman spectrum, enabling the detection of single labels even in complex biological media.^61^

**Figure 4:**
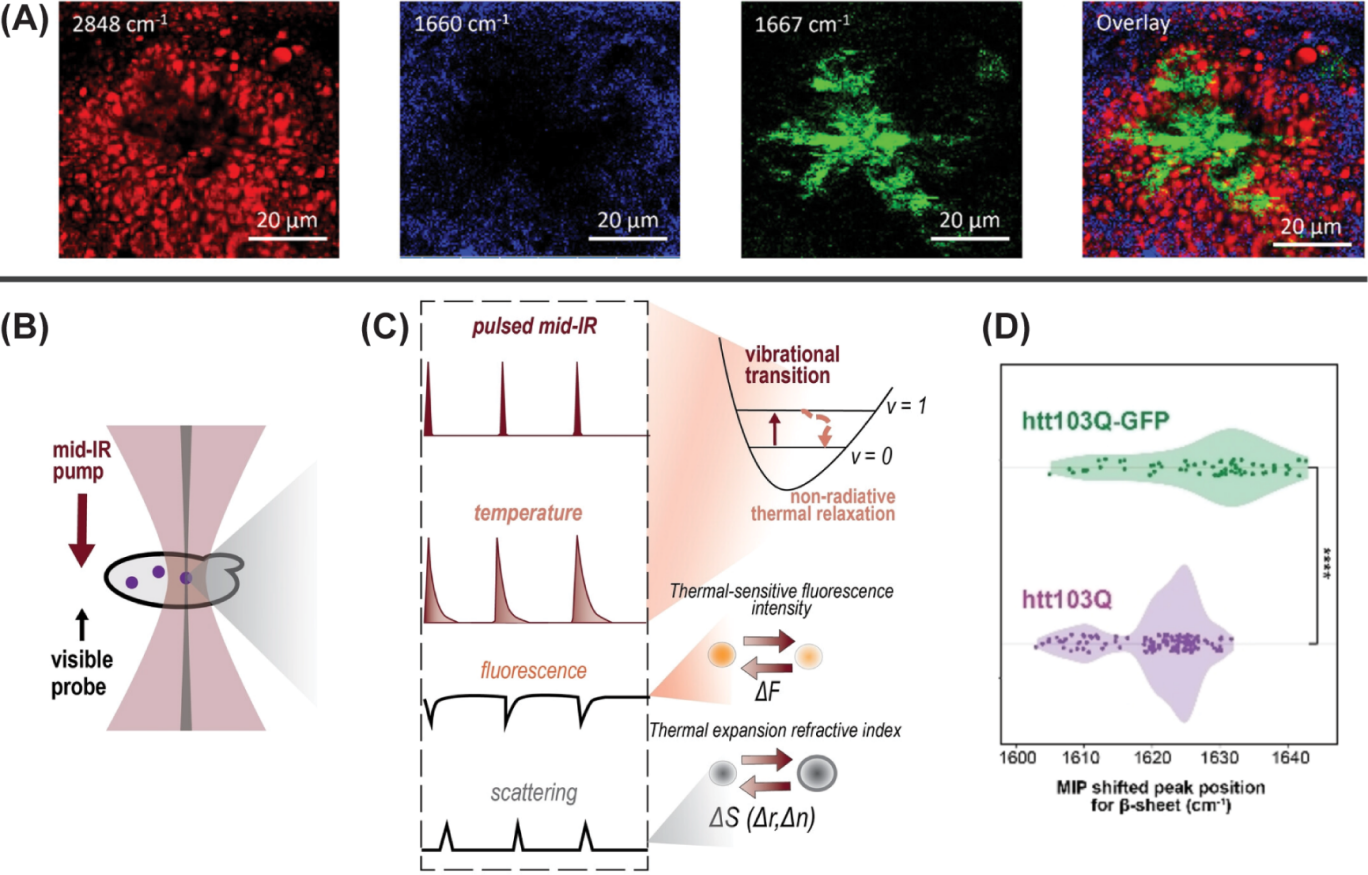
Vibrational optical microscopy: (A) Localization of lipids, proteins, and amyloid fibrils in amyloid plaques via hyperspectral Raman imaging at 2848 cm^–1^ (red), 1660 cm^–1^ (blue), and 1667 cm^–1^ (green). Reproduced from ref 69. Available under a CC BY 4.0 license. Copyright 2023 Mrđenović et al. (B) Schematic overview of OPTIR in counterpropagating geometry and (C) further details of signal generation. The photothermal response to incident IR light is detected via scattering of the incident visible light, while fluorescent probes can be used simultaneously to guide spectral assignment. (D) Aggregate OPTIR peak shift data for htt103Q with (green) and without (purple) GFP tagging. The presence of GFP significantly impacts the frequency of the fibril β-sheet peak (*p* = 3.18 × 10^–6^), indicating that GFP labeling can alter fibril structure. Panels B–D reproduced with permission from ref 70. Copyright 2024 Wiley-VCH GmbH.

### Infrared Spectroscopic Imaging

While the resolution of IR spectroscopic imaging is inherently lower than that of RSI due to the diffraction limit, it is still widely used in tissue analysis.^76^ Infrared microscopy in combination with deep convolutional neural networks has proven capable of label-free detection of Aβ in AD brain tissue with the same accuracy as gold-standard immunohistochemistry approaches.^77^ Beyond detection, IR spectroscopic imaging is also capable of differentiating between Aβ plaque types in AD^78^ or between two different amyloid types, both involved in cardiac amyloidosis.^79^ Both early work by Röhr et al. and more recent work by Holcombe et al. provide evidence for varying levels of antiparallel β-sheet structures in AD brain tissue sections, linking *in vitro* and *ex vivo* Aβ aggregation models.^27^ As antiparallel structures are more commonly associated with transient intermediate species hypothesized to be more toxic than fibrils in AD models, their discovery of plaques with significant populations of antiparallel sheets suggests that subtyping plaques based on their secondary structure rather than overall morphology may provide new insight into different stages or presentations of AD.

### Optical Photothermal Infrared Imaging

When IR light is focused onto a sample, the sample undergoes thermal expansion if the IR wavelength is resonant with a vibrational mode. This photothermal expansion also changes the refractive index of the sample. In OPTIR, also called mid-IR photothermal (MIP) microscopy, an IR pulse is used to induce the photothermal response, which is then probed with a short-wavelength visible pulse to measure changes in transmittance, refraction, or scattering (Figure 4B,C). The spatial resolution of OPTIR is approximately 10× better than conventional IR imaging, as it is determined by the diffraction limit of the visible probe pulse, not the IR excitation pulse.^80,81^ To overcome the challenge of identifying and isolating amyloid signals from those of other endogenous proteins and biomolecules, Prater et al. demonstrated that epifluorescence and OPTIR could be integrated into a single FL-OPTIR instrument.^82^ By treating tissue samples with fluorescent antibodies selective for amyloid plaques, they achieved submicron imaging of Aβ deposits in fixed cells. Guo et al. utilized a similar fluorescence-guided OPTIR setup to determine the secondary structural composition of plaques in a live yeast cell model of Huntington’s disease.^70^ Instead of using fluorescent antibodies, they tagged huntingtin (htt) variants with green fluorescent protein (GFP). However, they also demonstrated the label-free identification of protein aggregates. Comparison of tagged and untagged spectra revealed that the inclusion of a fluorescent tag perturbs the secondary structure of the aggregates, which highlights the need to understand how non-native labels may alter the already diverse array of structures formed by amyloid proteins (Figure 4D). They also discovered that yeast prion alters the structure of the htt aggregates, shifting them from small, nontoxic β-sheet aggregates to larger aggregates with a β-sheet core and α-helical shell. The combined spatial and structural resolution of OPTIR has also been employed to understand cross-seeding of amyloids in cells. Zhan et al. found that not only can α-syn coaggregate with tau, but the resulting copolymers are most effective at seeding pure α-syn aggregation, leading to aggregates with elevated β-sheet composition and phosphorylation levels.^83^ Recent work by de Oliveira et al. used OPTIR to investigate vascular deposits of Aβ associated with cerebral amyloid angiopathy (CAA).^84^ Despite the significant overlap between CAA and AD, they found that the vascular aggregates show an enhanced antiparallel β-sheet structure compared to the parallel β-sheet structure in AD plaques. They also found that the antiparallel structures are correlated with an increase in colocalized lipids, suggesting a lipid-mediated aggregation pathway. Thus, techniques such as OPTIR may provide new insights into the role of coaggregation, cross-seeding, and lipids in the molecular heterogeneity of amyloid diseases.

## Probe-Based Vibrational Imaging

To overcome the diffraction limit, vibrational spectroscopies can be coupled with probe-based imaging techniques to improve spatial resolution. In this section, we discuss probe-based imaging with both IR and Raman spectroscopy.

### Atomic Force Microscopy-Infrared Spectroscopy

Atomic force microscopy infrared (AFM-IR) is perhaps the most common IR-based imaging technique. Like in the case of OPTIR, AFM-IR relies on measuring the photothermal response of a sample. However, AFM-IR employs an AFM cantilever tip to measure the thermal expansion of the sample as the wavelength of the IR beam is tuned, allowing an IR spectrum to be generated for the nanoscale sample region just under the tip.^87^ The exceptional spatial and structural resolution of AFM-IR shows great promise for resolving heterogeneities not just within amyloid samples^88^ but also within individual aggregates. Banerjee et al. showed that Aβ oligomers are structurally heterogeneous and that their heterogeneities are propagated to the fibrils. Surprisingly, they found that this can result in domains with distinct secondary structures even within a single fibril.^89^

AFM-IR has also found use in studying heterotypic amyloid fibrils formed via coaggregation or cross-seeding of Aβ and human amylin.^85^ By using native amylin and ^13^C-labeled Aβ, Baghel and Ghosh easily differentiated amide I signals arising from either species. Not only were they able to directly observe both ^12^C and ^13^C signals within signal fibrils, providing unequivocal evidence for mixed aggregation, but they could discern that coaggregation leads to a unique fibril polymorph not observed in either pure amylin or pure Aβ samples (Figure 5A,B). As growing evidence supports the colocalization or coaggregation of different proteins within amyloid plaques, AFM-IR is particularly well-suited to examine the structure of two or more proteins within mixed aggregates.

**Figure 5:**
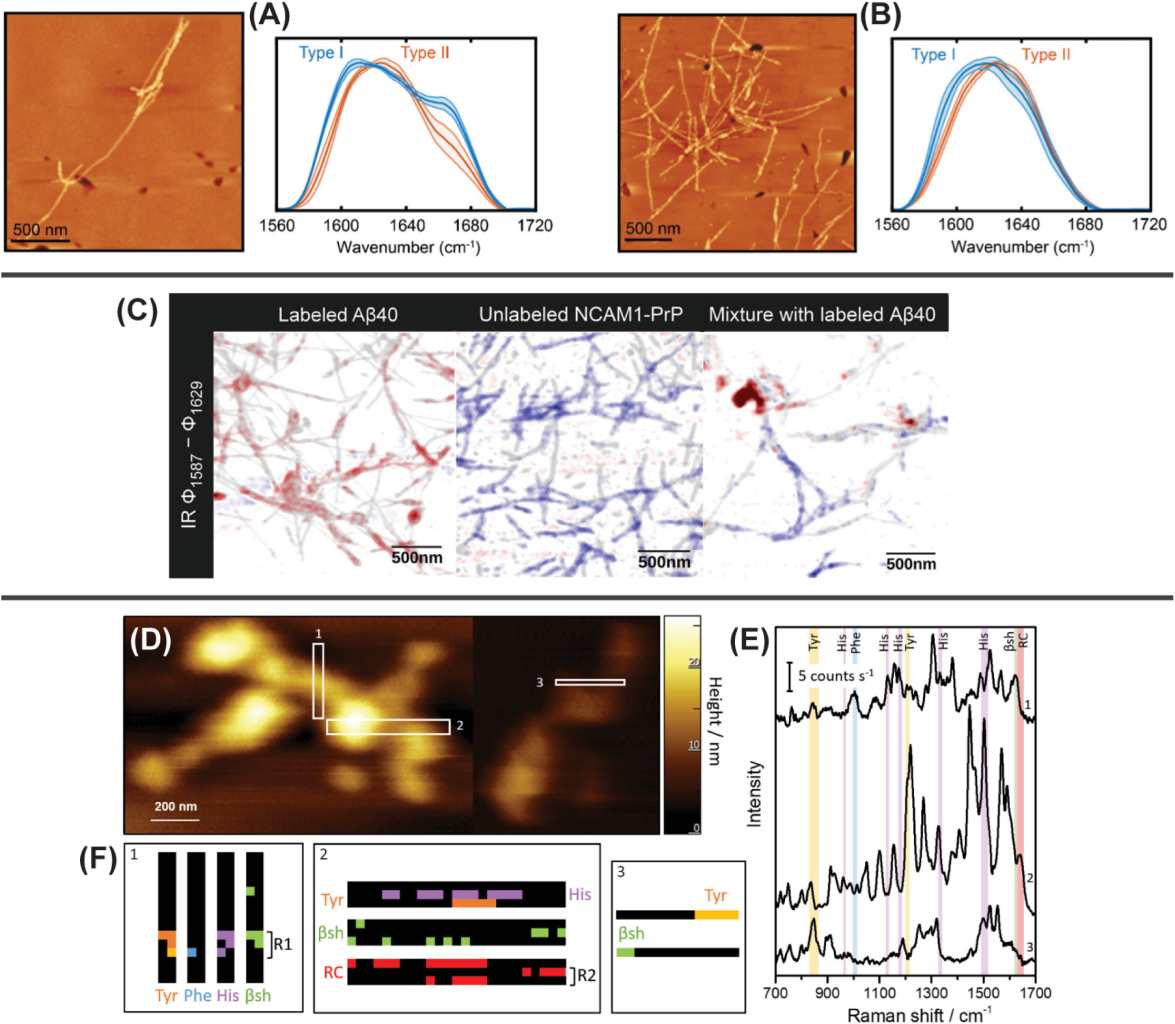
Probe-based vibrational imaging: (A) AFM image and corresponding AFM-IR spectra of ^13^C-labeled Aβ_42_ coaggregated with amylin after a 72 h aggregation period. Two distinct fibril morphologies are observed, but both contain asymmetric amide I bands, indicating the formation of heterotypic fibrils containing both Aβ_42_ and amylin. Coaggregated fibrils appear to form a unique polymorph distinct from either pure Aβ_42_ or pure amylin. (B) AFM image and corresponding AFM-IR spectra of ^13^C-labeled Aβ_42_ cross-seeded with amylin after a 72 h aggregation period. The more symmetric line shape matches that of pure Aβ_42_, suggesting that amylin is ineffective at seeding Aβ_42_ aggregation. Panels A-B are adapted from ref 85. Copyright 2025 American Chemical Society. (C) sSNOM difference images of ^13^C^15^N-labeled Aβ40 (left), unlabeled inhibitory peptide NCAM1-PrP (middle), and a mixed sample (right) indicate that NCAM1-PrP either colocalizes with or dissolves Aβ40 fibrils. Adapted from ref 81. Available under a CC BY 4.0 license. Copyright 2023 Paul et al. (D) AFM images of L34T Aβ_42_ fibrils with (E) average TERS spectra obtained from the regions highlighted by white boxes. (F) TERS maps showing the spatial distribution of Tyr, Phe, and His residues within each box, as well as regions of β-sheet (βsh) and random coil (rc) structures. Panels D–F reproduced from ref 86. Copyright 2024 American Chemical Society.

### Scattering-Type Scanning Near-Field Optical Microscopy

Scanning near-field optical microscopy (sSNOM) is complementary to AFM-IR and can be integrated into the same instrument. While AFM-IR measures the mechanical response of the sample upon IR irradiation, sSNOM measures IR light scattered by the AFM tip. Using interferometric detection, information about the absorptivity and refractive index of the sample is obtained.^81^ Unlike AFM-IR, sSNOM can be performed in two modes. A monochromatic source can be used for rapid imaging, as the tip is raster-scanned across the sample. Alternatively, the sample can be irradiated with a broadband light source followed by a traditional Fourier transform analysis to obtain a full IR spectrum with nanometer spatial resolution (nano-FTIR). As with AFM-IR, sSNOM and nano-FTIR are capable of resolving the molecular structures of mixed protein aggregates. Paul et al. used both modes to study the interaction of Aβ with an inhibitory peptide, NCAM1-PrP.^81^ They first collected nano-FTIR data to characterize the amide I and amide II frequencies of unlabeled Aβ, ^13^C^15^N-labeled Aβ, and NCAM1-PrP. While ^13^C^15^N labeling resulted in only a ∼40 cm^–1^ shift of the amide I mode, an accompanying 20 cm^–1^ shift of the amide II mode helped them confidently distinguish between labeled and unlabeled Aβ fibrils in a mixed sample. From the nano-FTIR spectra, they were able to identify two frequencies for sSNOM imaging: 1629 cm^–1^, which was near the amide I maximum for both unlabeled Aβ and NCAM1-PrP, and 1587 cm^–1^, which is near a minimum for the unlabeled peptides but a maximum for ^13^C^15^N-labeled Aβ. Thus, by subtracting sSNOM images collected at the two frequencies, they were able to observe spatial distributions of unlabeled and labeled peptides in each sample (Figure 5C). From the images, they found that preformed Aβ fibrils disappeared after the addition of NCAM1-PrP, suggesting that the inhibitor can restructure or dissolve fibrils.

### Tip-Enhanced Raman Spectroscopy

Tip-enhanced Raman spectroscopy (TERS) has emerged as a high-resolution alternative for hyperspectral imaging of amyloid fibrils. TERS combines the advantages of surface-enhanced Raman spectroscopy and scanning probe microscopy by focusing a laser beam onto a tip coated with a plasmonic metal. The signal is observed only from directly under the tip, where Raman scattering is enhanced by up to 10^11^, resulting in nanometer spatial resolution even under ambient conditions.^90^ Most probe-based techniques, including AFM-IR and sSNOM, require samples to be dried before they can be imaged; this has traditionally been true for TERS as well.^91,92^ Recently, however, Lipiec et al. pioneered the application of TERS imaging to aqueous amyloid samples, demonstrating their ability to characterize single aggregates throughout the aggregation pathway of Aβ.^93^ They observed antiparallel β-sheets in both oligomers and protofibrils that rearranged into a parallel alignment with the final fibrils. They also found small aggregates with antiparallel structures near the surface of fibrils, which they attributed to secondary nucleation. Surprisingly, when they compared TERS spectra obtained in aqueous versus dry conditions, they found that the liquid samples resulted in higher signal-to-noise ratios and better reproducibility. Others have continued to advance the application of TERS in aqueous samples, even resolving the spatial distribution of aromatic side chains within aggregates (Figure 5D–F).^86^ These studies position TERS as an incredibly powerful approach for studying single amyloid aggregates in their native environments.

## Summary and Future Outlook

Vibrational spectroscopy and imaging have proven to be powerful tools for unraveling the complex mechanisms and structures involved in amyloid aggregation. In this perspective, we review recent progress in addressing various challenges, ranging from improving structural resolution to multianalyte heterotypic systems and even measurements in tissues.

Raman and IR spectroscopy serve as the foundation for most of the more advanced techniques that we have discussed and, even in their most fundamental forms, are still actively used to investigate amyloid heterogeneity. With intrinsically nonperturbative structural sensitivity, these techniques can be used to track aggregation via redshifting of the amide I mode^63^ and differentiate between aggregate structures using a combination of the three major amide modes.^35^ Their ability to resolve structural heterogeneities can be enhanced using site-specific labels such as isotopes or UAAs. Isotope labeling of backbone amide groups is particularly appealing for shorter peptides, as isotopic substitution is nonperturbative and enables direct measurements of secondary structure for individual residues.^53^ The improved sensitivity of 2D IR spectroscopy, as well as the ability to optimize the 2D IR pulse sequence to selectively suppress unwanted background signals,^65^ makes it the best technique for these studies. However, IR spectroscopies suffer from strong water absorption in the amide I region, which requires samples to be prepared in deuterated buffers and hinders their use in biological environments. Recently, the Hunt group pioneered the collection of 2D IR spectra in H_2_O by taking advantage of water’s relatively weak molar extinction coefficient and short vibrational lifetime to minimize the water background.^94^ Their work has enabled direct observation of insulin aggregation in water,^95^ although single isotopes still seem to be out of reach. Alternatively, Wat et al. demonstrated a novel “reverse-labeling” scheme, which allows selective labeling of a single residue type at relatively low cost.^96^ Cells are grown in ^13^C-enriched media, with a specific ^12^C-amino acid added when the expression is induced. This produces proteins in which only residues of that type have ^12^C-labeled carbonyls while the rest of the protein is uniformly ^13^C-labeled. This approach is particularly promising for understanding the role of the cellular environment on amyloid aggregation, as they were able to detect the ^12^C-labeled residues even in live bacterial cells without needing to purify the protein from the cellular background.

Unlike IR spectroscopy, Raman spectroscopy does not suffer from significant water backgrounds but generally lacks the sensitivity to observe single isotope labels, so uniform or segmental isotope labeling is more commonly employed for Raman spectroscopy and imaging. Alternatively, UAAs with non-native functional groups can also be used as site-specific vibrational probes. UAAs can be genetically encoded for cellular protein expression, enabling their use in larger proteins, and are compatible with biological environments, as their functional groups are chosen to appear within the cell-silent region of the vibrational spectrum, allowing them to be observed with no spectral interference from other biomolecules.^61,66,97^ These probes are particularly useful for Raman studies, as Raman can observe both the UAA modes and the native protein amide modes simultaneously, allowing the signals to be correlated. While more perturbative than isotopes—a critical concern for amyloid studies due to the extreme sensitivity of aggregate structures to even small changes in conditions—these functional groups are much smaller than the fluorescent tags and spin labels used for other techniques and remain a promising approach for studying amyloid aggregation in complex cellular environments.

Even without labeling, researchers have made significant advances in resolving spectral contributions from heterogeneous mixtures with techniques such as TDS analysis and IR-DOSY. TDS, in particular, can provide incredibly sensitive measurements of α-helical length^98^ and β-strand organization.^38,48,49^ Although powerful as a label-free technique, it can also be combined with site-specific labeling to track changes in TDS at a single residue throughout the amyloid aggregation process,^39^ enabling new insights into transient oligomeric structures that may be inaccessible by other techniques. Alternatively, IR-DOSY’s ability to physically separate species by size while maintaining structural information is an elegant approach for studying complex mixtures of biomolecules.^37,43^ However, more advanced data analysis techniques are needed to address the challenge of applying IR-DOSY to rapidly evolving sample mixtures, such as during the active aggregation of amyloid monomers into oligomers, protofibrils, and fibrils.

While solution-based vibrational spectroscopies can provide a wealth of information about amyloid structure, coupling these techniques with imaging modalities adds an extra dimension for resolving heterogeneities. RSI is a robust, versatile tool capable of imaging amyloids in both cells and *ex vivo* tissues and can be implemented on labeled samples, similar to solution-phase Raman. OPTIR has emerged as a complementary approach, enabling IR imaging with the same spatial resolution as RSI. To capitalize on the strengths of both techniques, a hybrid instrument capable of simultaneous OPTIR and RSI measurements has been demonstrated recently.^80^ Spatial resolution can be further improved by coupling vibrational spectroscopies to probe-based imaging techniques. AFM-IR, sSNOM, and TERS all circumvent the diffraction limit by measuring the signal generated only in the vicinity of an AFM tip. With nanometer spatial resolution, these techniques can examine protein–protein interactions within mixed aggregates^85^ and tease out structural heterogeneities even within individual fibrils.^89^ However, the trade-off for improved spatial resolution is a loss of dynamics, as samples typically must be dried before measurement. In recent progress, TERS has been demonstrated on aqueous samples^86,93^ but still lacks the ability to temporally resolve structural changes.

Looking into the future, we anticipate that the integration of vibrational spectroscopies with imaging techniques will continue to expand. Nonlinear vibrational spectroscopies provide many advantages in terms of improved sensitivity and information content but are challenging to couple with imaging modalities. While nonlinear Raman techniques such as SRS and CARS have been successfully implemented in optical microscopes and are frequently applied to biological samples,^72,73,75^ nonlinear IR techniques lag behind. Given the distinct advantages of 2D IR spectroscopy for resolving weak protein signals and providing label-free resolution of polymorphs via TDS and crosspeak analysis, 2D IR imaging could be a powerful tool for unraveling amyloid aggregation pathways. 2D IR microscopy has been realized but not widely adopted due to challenges in its implementation.^99^ Despite these challenges, Dicke et al. demonstrated the use of 2D IR microscopy to study structural heterogeneities within pancreatic tissues obtained from mice.^100^ Recently, AFM 2D IR has also been demonstrated for the first time,^101^ although it has not yet been applied to protein analysis. Additionally, machine learning algorithms show great promise for aiding the interpretation of complex vibrational spectra. Machine learning algorithms have been used to recognize signatures of drugs bound to blood serum proteins in 2D IR spectra,^102^ identify α-syn aggregates at different stages of aggregation in Raman spectra,^103^ and even characterize amyloid subtypes in human kidney tissue.^104^ Ultimately, we believe that vibrational techniques will continue to grow into an essential tool for unraveling the complexities underlying amyloid-related diseases. Their compatibility with a wide range of sample conditions and ability to provide structural details for aggregates of various sizes and at various stages of aggregation will propel the field toward a deeper understanding of amyloid disease and guide the strategic design of targeted therapeutics.
